# Synthetic data for annotation and extraction of family history information from clinical text

**DOI:** 10.1186/s13326-021-00244-2

**Published:** 2021-07-14

**Authors:** Pål H. Brekke, Taraka Rama, Ildikó Pilán, Øystein Nytrø, Lilja Øvrelid

**Affiliations:** 1 Oslo University Hospital, Rikshospitalet, Department of Cardiology, Sognsvannsveien, Oslo, Norway; 2 University of North Texas, Department of Linguistics, Discovery Park, Denton, TX USA; 3 University of Oslo, Department of Informatics, Blindern, Oslo, Norway; 4 Norwegian University of Science and Technology, Department of Computer Science, Trondheim, Norway

**Keywords:** Natural language processing, Synthetic data, Corpus annotation, Family history, Heart disease

## Abstract

**Background:**

The limited availability of clinical texts for Natural Language Processing purposes is hindering the progress of the field. This article investigates the use of synthetic data for the annotation and automated extraction of family history information from Norwegian clinical text. We make use of incrementally developed synthetic clinical text describing patients’ family history relating to cases of cardiac disease and present a general methodology which integrates the synthetically produced clinical statements and annotation guideline development. The resulting synthetic corpus contains 477 sentences and 6030 tokens. In this work we experimentally assess the validity and applicability of the annotated synthetic corpus using machine learning techniques and furthermore evaluate the system trained on synthetic text on a corpus of real clinical text, consisting of de-identified records for patients with genetic heart disease.

**Results:**

For entity recognition, an SVM trained on synthetic data had class weighted precision, recall and F_1_-scores of 0.83, 0.81 and 0.82, respectively. For relation extraction precision, recall and F_1_-scores were 0.74, 0.75 and 0.74.

**Conclusions:**

A system for extraction of family history information developed on synthetic data generalizes well to real, clinical notes with a small loss of accuracy. The methodology outlined in this paper may be useful in other situations where limited availability of clinical text hinders NLP tasks. Both the annotation guidelines and the annotated synthetic corpus are made freely available and as such constitutes the first publicly available resource of Norwegian clinical text.

## Background

Progress in the field of clinical Natural Language Processing (NLP) is currently limited to a large extent by the availability of annotated clinical text. Such text originates in the (electronic) health record (EHR), and access to and use of the EHR is governed by strict data privacy and health service regulations, which usually restrict secondary use. Among notable exceptions are anonymized health record texts published as part of the *i2b2* challenges [1] and the CLEF corpus [2]. For languages other than English, however, the situation is even more difficult, and despite notable annotation efforts [3], the underlying corpora are largely unavailable [4]. One alternative in light of this situation is to investigate possibilities for the use of synthetic data in the development of clinical NLP tools [5–7].

Modern NLP methods require manually annotated data, and the design of annotation guidelines is crucial for consistent and high quality data suitable for machine learning and classification. Clinical texts are radically different in form and function from other biomedical texts: They are communicative, conveying information between health service providers, terse (in that the patient is implicit), and very specialized according to the role of the narrative and profession of the author [8, 9]. Development of annotation guidelines is a time consuming process which in the case of clinical data often also requires access to domain experts (clinicians). The question of how to involve the clinician in the annotation process and make the best use of their domain knowledge is therefore highly relevant.

This article describes the systematic development of annotation guidelines for family history information in Norwegian clinical text. We make use of incrementally developed synthetic clinical text describing patients’ family history relating to cases of genetic cardiac disease. The domain expert is an integral part of this methodology and generates synthetic examples that challenge the guidelines and further participates both in the annotation and development of guidelines. In doing so, the domain knowledge of the clinician informs the annotation process systematically.

In the rest of the paper, we describe the methodology for corpus generation and annotation guideline design in more detail. We briefly present inter-annotator agreement based on the developed guidelines and results from machine learning experiments aimed at evaluating the validity and applicability of the purpose-made annotated corpus. We furthermore compare results on synthetic and de-identified electronic health records, and show that our system trained on synthetic text generalizes well to real, clinical text. The article is based on [10], however, crucially extends on the methodology first described there by applying it to annotation and processing of real, de-identified clinical text.

### Family history in clinical text

**Fig. 1:**
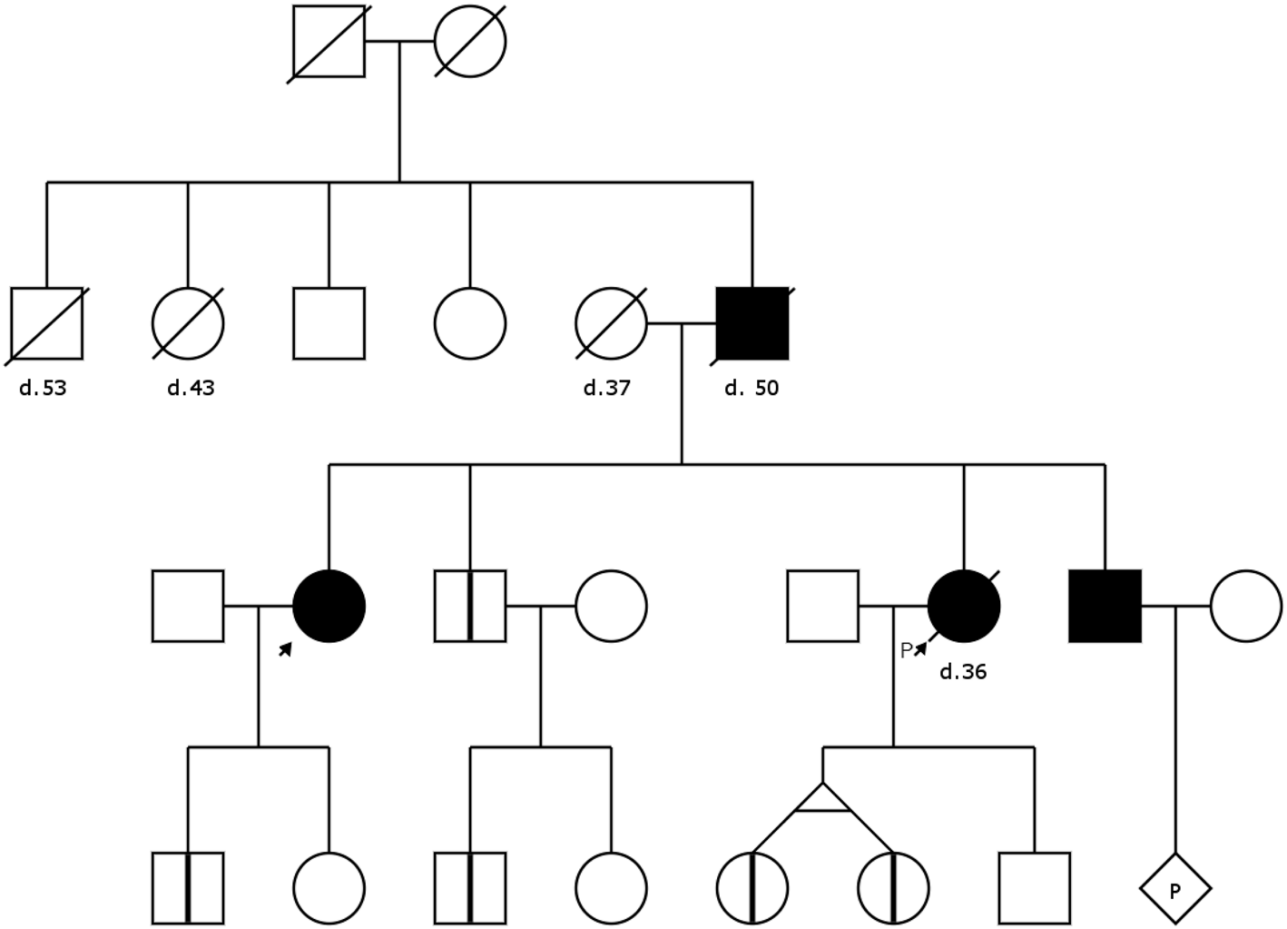
An example pedigree chart with a typical autosomal dominant inheritance pattern. Horizontal rows represent generations, lines represent relationships, lines of descent and sibship. Squares are male, circles female, and diamond shape is unknown gender. A symbol with a ‘P’ inside denotes a pregnancy. Diagonal lines through symbols denote deceased individuals and the text below their age at the time of death (eg. ‘d. 43’ means died when 43 years old). Filled symbols represent individuals with manifest disease, symbols with a vertical line are healthy gene carriers who may develop disease later. The small arrow denotes the current patient (“self”) and the arrow with the ‘P’ is the proband or index patient where the genetic analysis of the family started [11]

A family history is an important part of the medical record. It helps the clinician in identifying risk factors, in diagnosing conditions that have genetic components, and in identifying family members who should be offered genetic counselling or medical follow up. Specific patterns of disease or symptoms in a family suggest modes of inheritance, and could be helpful in the diagnosis of an unrecognised disease or syndrome. In the cases where a pathological mutation has already been identified, the pedigree is used to plan further genetic screening or counselling. Figure 1 shows an example pedigree with an autosomal dominant inheritance pattern.

For some diseases, the course of events in the patient’s family is important in judging the patient’s own risk of serious events. In patients with hereditary hypertrophic cardiomyopathy (HCM), the European Society of Cardiology recommends using an online risk calculator to estimate a patient’s 5 year risk of sudden cardiac death (SCD). Among the seven factors included in the underlying model – and a strong contributor to individual risk – is a history of SCD in first degree relatives [12]. The current work was motivated by a task of automating risk prediction for HCM patients seen in the outpatient clinic.

Family histories occur as descriptive text in the EHR, but acknowledging that computational reasoning about family history has substantial benefits in research, diagnosis and decision support, many tools have been developed for interactive pedigree input [13]. The underlying objective of our NLP challenge is to be able to infer the pedigree of a patient from text. However, even checking consistency of family history information represented in OWL proves to be a challenge [14]. A potential outcome of our work would be to transform statements about pedigree into tabular formats directly usable in risk calculators and for bioinformatics applications like genome-wide analysis [15].

### Previous work

There has been some previous work aimed at extracting family history information from clinical text. [16] annotate 284 sentences from the publicly available MTSamples corpus of synthetically produced English clinical text for information about family members and clinical observations with some additional attributes (vital status, negation and age of death). However, they do not provide any measures of inter-annotator agreement. [17] compared the information contained in structured and free-text descriptions of family history information and found that the free-text descriptions were more comprehensive.

In another work, [18] developed a pipeline of rule based systems to detect family members and diagnosis concepts and then assign the family diagnosis to a specific family number. The authors run standard NLP tools such as sentence splitter and part-of-speech taggers on discharge summary notes. The pipeline system is related to [19] in only identifying diagnosis concepts that are present in standard medical dictionaries and do not perform relation extraction as performed in this paper.

Major past work on relation extraction from clinical reports is based on rule based systems [20] and machine learning methods (based on multi-class SVMs) [21, 22]. Our work in this paper is closest to the work of [21] who manually annotated cancer narratives for entities and relations, and then trained and tested a one-vs-rest SVM classifier for training and testing. In this paper, we employ widely used features in general purpose named entity recognition [23, 24] to train SVM models for family history extraction.

More recently (and contemporaneous with this work), one of the BioCreative/OHNLP shared tasks featured a family history extraction task for English clinical text [25]. The annotation scheme employed in their work is very similar to the one presented here, however, they limit the types of family members extracted and do not explicitly annotate temporality. The corpus employed in the task contains a total of 149 clinical notes annotated for a number of clinical entities related to family history. The entities furthermore had several attributes. The annotated entities were Family member (with attributes Side, Blood and Adopted), Observation (with attributes Negation and Certainty), Living Status (with attributes Alive and Healthy) and Age (with attributes Type, Range and Value). The best performing system in the shared task achieved an overall F-score of 88.6 for the task of identifying Family Member and Observation entities only (Track 1). For the full extraction task (recognizing Family, Observation, Age, Living Status and attributes) the best performing system reached an F-score of 57.1.

## Methods

### Incremental annotation guideline and synthetic corpus development

With the goal of extracting family history information from Norwegian clinical text, and real health records being unavailable at the start of the project, we developed a methodology for incremental development of annotation guidelines in tandem with the production of a synthetic text corpus.

The synthetic corpus was produced by a cardiologist with extensive clinical experience, and expertise in genetic heart disease. The statements produced correspond to a small part of the patient record concerning the patient’s family history. Descriptions were inspired by web searches for “autosomal dominant pedigree”, where descriptions of parts of the resulting pedigrees were described while assigning realistic but invented medical events. No actual patient histories are reproduced, but coincidental similarities to real events must be expected.

The guideline developers consisted of a clinician and three computational linguists and/or computer scientists. We usually maintained two roles: The clinician would produce a set of representative sentences and along with one of the others propose an annotation scheme for these. Then, the clinician would annotate while another independent person not involved in the design of the annotation scheme would make an *independent annotation*. The results were compared and discrepancies were recorded. We (sometimes artificially) could identify both *semantic* and *pragmatic* discrepancies. Semantic discrepancy would signify a misunderstanding of the underlying domain and required amending the ontology, whereas the pragmatic discrepancy would uncover an underspecified or incomplete annotation rule which could be further specified by adding more examples to the corpus.

**Fig. 2:**
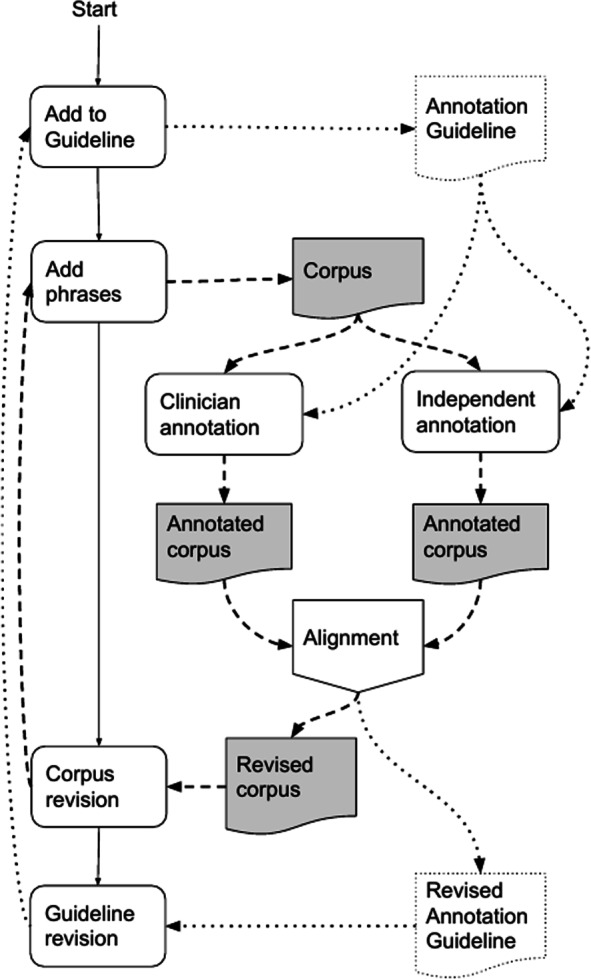
Incremental development of corpus and annotation guidelines

Figure 2 shows the double loops of corpus production and guideline development. As shown, the family history statements were produced iteratively. In the initial round, the clinician was asked to produce a set of representative statements about SCD-related family history.

Example 1 below shows a sentence from the corpus.

#### **Example 1**

(1) Indekspasienten er hans onkel på farssiden, som hatt hjertestans og fått implantert ICD. Index-patient is his uncle on father’s-side, who had cardiac-arrest and had implanted ICD.‘The index patient is his uncle on the father’s side, who had cardiac arrest and implanted ICD.’

Following the initial iterations and discussions with the clinician the need to account for i) relations to groups of family members, ii) temporal statements, and iii) negation emerged. During this iteration the clinician was therefore tasked with the generation of statements that challenged the current guidelines, whilst still producing representative family statements. Example 2 shows a sentence containing a temporal statement.

#### **Example 2**

(2) Han har kjent hjertebank de siste fire-fem månedene. He has felt heart-palps the last four-five months‘He has been feeling heart palpitations during the last four-five months.’

After arriving at a fairly stable set of guidelines, a large portion of the data set (320 sentences) was doubly annotated. Following this, disagreements were resolved in a round of consolidation between the annotators. The final portion of the data set (91 sentences) was then annotated doubly and the resulting inter-annotator agreement on these data sets is reported below in “namerefsec:annotguideAnnotation guide-line” section.

### Dataset of de-identified clinical notes

With the approval of the regional medical ethics board, we got access to de-identified medical records for 350 patients with genetic heart disease followed at Oslo University Hospital. Records were manually checked for personally identifying data by a cardiologist before release for NLP use. The dataset comprised 2,276 outpatient notes.

All annotation was performed using the Brat web-based annotation tool [26]. The data was automatically segmented and tokenized prior to annotation.

### Annotation guidelines

**Fig. 3:**
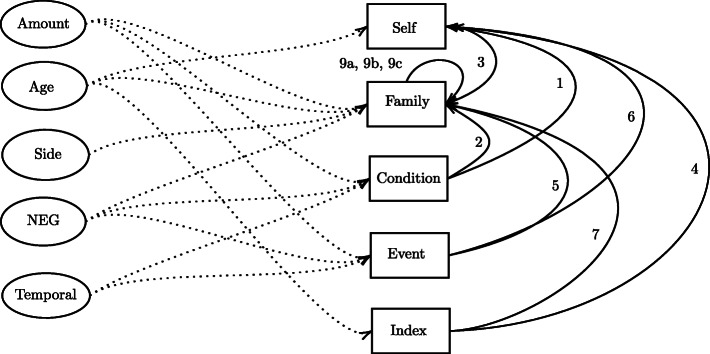
Schematic diagram showing the possible relations between entities. The different relations are marked with a number to avoid cluttering. Holder: 1, 2, 4, 5, 6, 7, 8; Modifier: Dotted lines; Related to: 3, 9a; Subset: 9b; Partner: 9c

The annotation guidelines have been made publicly available and are described in [10]. The following section presents an overview of the annotation guidelines developed along with the synthetic corpus. The annotation of the corpus distinguishes semantically relevant clinical *entities* and shows how these relate to each other in the text via a set of *relations*. Figure 3 shows a graphical overview of the annotation schema, where rectangles indicate core clinical entities, ovals indicate modifier entities, and all possible relations are indicated by directed arcs.

### Clinical entities

Clinical entities are continuous text spans marked with one of the following entity types:

- Family describes various family member types (e.g. *onkelen* ‘the uncle’, *bestefar* ‘grandfather’).
- Self is used only for the patient under consideration (e.g. *pasienten* ‘the patient’, *hun* ‘she’).
- Index entities designate the property of being the index patient or *proband*, i.e. the first identified family member with disease (e.g., *indekspasienten* ‘the index patient’).
- Condition entities describe a range of clinical conditions such as diseases (*koronarsykdom* ‘coronary disease’), diagnoses, various types of mutations, test results (*testet negativt* ‘tested negative’), treatments (*hjertetransplantert* ‘heart-transplanted’), and vital state (*død* ‘dead’, *frisk* ‘healthy’).
- Event entities describe clinical events (e.g. *hjertestans* ‘cardiac arrest’ and *synkope* ‘syncope’).

The distinction between conditions and events relate to the temporal extension of the entity described: an event is something that happens and then is over, but a condition is a prolonged state of the patient, for instance, the patient has a heart attack (Event), but from this point on she is considered to have heart disease (Condition).

In addition to the main clinical entities described above, the annotation guidelines also distinguish a set of modifier entities that further describe the clinical entities for a number of properties that are relevant for semantic interpretation of family history information:

- Side entities describe the side of the family and thus modify Family entities (e.g. *farssiden* ‘paternal side’).
- Age entities describe the age of a family member e.g., 40 år gammel ‘40 years old’.
- Negation entities mark lexical items that signal negation, so-called *negation cues* in the terminology of [27]. These may be negative adverbs, such as e.g., *ikke* ‘not’, *aldri* ‘never’, or negative determiners/pronouns *ingen* ‘nobody’. Note that in contrast to [27], we do not annotate morphological negation cues (e.g. ***im***-*possible*). In this version of the guidelines, we treat negation as encompassing uncertainty. The main reason for this is that just like the presence of negation, it marks missing information that should not be included in the family history.
- Amount modifiers describe quantifiers that describe numerical properties of clinical entities, e.g. *to* ‘two’, *mange* ‘many’.
- Temporal modifiers typically position Condition/Event entities in time, e.g. *i sommer* ‘this summer’, *for tre år siden* ‘three years ago’. These are similar to temporal expressions (so-called *timexes*) in previous temporal annotation schemes [28, 29].

### Family history relations

**Fig. 4:**
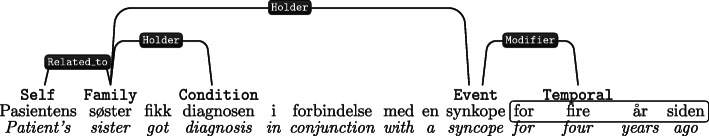
Annotation of clinical entities and relations for an example sentence from the corpus
- Modifier relations hold between modifier entities (e.g. Side, Negation) and clinical entities (e.g. Family, Condition).
- Related_to relations specify relations between family members and always hold between entities of the Family type.
- Subset relations specify relations between family members, where one is a subset of the other, e.g. in statements such as *Hun har to brødre, den ene har mutasjonen* ‘She has two brothers, one of them has the mutation’, where *den ene* ‘one of them’ would be connected to the Family entity *brødre* ‘brothers’ with a Subset-relation.
- Partner relations specify relations between entities of the Family type, used to identify couples (husbands and wives, civil partnerships) that are able to provide offspring. The assumption is no kinship.

In addition to the clinical entities described above, we further annotate a number of relationships between entities in our annotation scheme. Figure 4 shows a fully annotated example containing entities and their relations for a sentence from the corpus. The relations are binary relations of the following types:

- Holder relations are always between Condition/Event entity on the one hand and its holder, a Family/Self/Index entity.

## Results

The annotated synthetic corpus contains 477 sentences and 6030 tokens. In Table 1 we present the distribution of the entities and relations in the corpus. We see that Condition and Event entities are fairly equally distributed in the corpus. Temporal modifiers span more than one word in a majority of cases. Whereas Holder-relations are the most common type of relation in the corpus, there are only 14 cases of the Partner relation.

**Table 1:** Distribution of entities and relations in the synthetic data annotated by the clinician. The Spans column shows the number of entities that span across words. Both the entities and relations are sorted in decreasing order of number of occurrences

	Number	Spans
Entities		
Family	1704	96
Condition	681	135
Event	542	115
Self	509	–
Amount	273	9
Temporal	214	178
Negation	131	33
Age	57	34
Side	36	3
Index	7	–
Relations		
Holder	880	–
Modifier	687	–
Related_to	389	–
Subset	108	–
Partner	14	–

Inter-annotator agreement is reported in detail in [10]. Briefly, we found that IAA scores improved between rounds of guideline improvement and annotations, with some remaining discrepancies between the clinican’s annotation (treated as gold standard) and the second annotator. Some of these are what we termed semantic discrepancies in “Methods” section above, annotation decisions that require domain knowledge. There are also examples where additional distinctions could be added to the guidelines, in particular with respect to annotation of temporal and negation-related information, both examples of complex annotation tasks by themselves. Overall, precision, recall and micro F_1_-score for agreement between the clinician and second annotator on entities spans and their labels reached 0.821, 0.797 and 0.809, respectively.

### Preliminary experiments on synthetic data

In this section, we perform entity classification and relation extraction experiments to verify the viability of our annotation. The domain expert annotated dataset has 477 sentences. We train and test a SVM model on the data with five-fold cross-validation.

### Entity detection

In this experiment, we trained and tested a linear classifier (SVM model) for entity classification. We treat entity classification as a multi-class classification problem where there are 11 classes including the “O” label that denotes unmarked lexical units. Our model is a linear SVM model that is trained on the following features:

- Lexical: Current word, words in a context window size of 2.
- Universal POS tags: Current word, words in a context window size of 2.
- Entity tags: The two previous entity tags where the model uses the gold entity tags to train but uses the previous predicted entity tags to predict the current tag.

We also experimented with lowercasing a word and orthographic features such as prefixes and suffixes of length 3 which did not improve the performance of the SVM model. For comparability with previous literature, we also trained a model using Conditional Random Fields (CRF) [30] with the sklearn-crfsuite Python library^1^. Unlike the SVM, which classifies entity labels for single tokens, the CRF predicts a sequence of entity labels for a whole sentence. For the CRF model, we employed the default training algorithm, gradient descent with the Limited-memory Broyden–Fletcher–Goldfarb–Shanno method, and 0.1 as coefficient for Elastic Net (both L1 and L2) regularization. The differences between the CRF and the SVM are significant at 0.05 level across precision, recall and F_1_ scores in the setup including ’O’. In the setup excluding ’O’, the difference is significant for F_1_ and precision but not for recall. The *p*-values were obtained with a t-test for paired samples on the 5 cross-validation fold results.

Our baseline is a rule-based approach where a dictionary is created by collecting words and their entity labels from the training data. (For the synthetic dataset, a separate dictionary is created for each cross-validation fold.) This dictionary baseline classification chooses the most frequent entity label for each word in the dictionary based on the training data, while words not appearing in the dictionary, are tagged as “O”.

We evaluated the performance of our models using weighted F_1_ score to account for class imbalance. On average, these feature templates yielded 5000 features across the five cross-validation experiments. CRF results are reported on the same features and random-split folds of the data. All the Universal POS tags are obtained through the CoNLL17 Baseline model [31] trained on the publicly available Universal Dependencies Norwegian Bokmål treebank [32]. The results of our experiments are given in Table 2, where we report scores both including and excluding the “O” label.

**Table 2:** The average of the weighted F_1_-scores across the five folds. On an average, there are 6030 training instances and 1507 test instances

	Including “O”			Excluding “O”		
System	Precision	Recall	F_1_-score	Precision	Recall	F_1_-score
Dictionary baseline	0.721	0.624	0.638	0.558	0.766	0.629
SVM	0.843	0.843	0.841	0.781	0.738	0.756
CRF	0.831	0.816	0.817	0.704	0.76	0.719

The SVM models were trained and tested on the whole of the data annotated by the annotator with medical knowledge. The SVM model performed better than the two baseline models across most measures. Although not entirely comparable given the difference in the nature of the prediction task, CRF results were overall rather similar, but somewhat lower than the performance scores of the SVM. The SVM model made errors at distinguishing Condition entities from Event entities and Age from Temporal entities. Most of the errors occurred when the SVM model misclassified the rest of the classes as “O”.

### Relation extraction

In this subsection, we performed a relation detection and classification experiment. In this experiment, we treat a relation defined between exactly two entities to belong to one of the six relations where five of them are given in Table 1 and the sixth relation is “No_Relation”. We train and test an SVM model in a five-fold cross-validation fashion. Apart from entity labels, we experimented with increasingly complex set of features:

- Lexical: Words belonging to the entities are treated as two separate features.
- POS tags: Universal POS tags of the entities’ lexical tokens as separate features.
- Dependency features: The dependency label of a entity word’s incoming arc as a feature.

If an entity is spanning across multiple words, we concatenate the per-word feature and treat them as a single feature when training and testing the SVM model. The results of the experiments are given in Table 3. Our results suggest that word based features themselves yield a performance which is close to the model with more complex features. Incremental inclusion of POS tags and dependency labels increases the performance of the SVM model, whereas the inclusion of predicted entity labels does not. Finally, including the gold standard labels improved the performance of the model.

**Table 3:** Average of the weighted F_1_-scores on five fold cross-validation

Features	Precision	Recall	F_1_-score
Words	0.716	0.732	0.719
+POS tags	0.73	0.738	0.731
+Dependency labels	0.743	0.746	0.743
+Entity labels (Predicted)	0.743	0.745	0.743
+Entity labels (Gold)	0.771	0.767	0.768

On an average, there are 5530 training instances and 1461 test instances

### Experiments on real data

We now go on to examine the question of how well the annotation and model developed using a synthetic corpus generalizes to real, de-identified clinical text. Importantly, this enables evaluation of the generalizability of the methodology above and the extent to which synthetic data can be useful in the case of family history extraction.

Sentences describing family relations from the outpatient notes were extracted using regular expressions matching a list of Norwegian lemmas for first-degree family entities^2^.

A random selection of 183 sentences from the outpatient notes were manually annotated by the same clinician who annotated the synthetic data, according to the current version of the annotation guidelines. As before, the data was processed using UDPipe [33], producing a tokenized, lemmatized, POS-tagged and dependency parsed version of the text for further processing.

The experiments with synthetic data suggest that the use of lexical features and POS features improved the performance of the SVM system as both entity recognition and relation extraction. In this section, we employ a SVM model trained on all of the synthetic data to test how well our annotation scheme fares on real data. An additional CRF model was not trained on this dataset given the results obtained on the synthetic data.

### Entity recognition

First, we predicted all the entity labels, with the results of these experiments given in Table 4. Each row shows the precision, recall, and F_1_-score and the number of test instances for each label. The test set is unbalanced. Therefore, we use class weighted evaluation metrics. The test set has 183 sentences and 3037 tokens. As expected, the majority of the tokens are labeled as “O”. The class weighted precision, recall, and F_1_-scores are given as the last rows of the Table 4, with SVM results followed by the dictionary baseline. The dictionary for this dataset was compiled using words from the whole synthetic dataset to ensure comparability with the SVM results. The F_1_-score is quite close to the average weighted F_1_-score reported on the synthetic dataset. The SVM classifier performs the best at classifying FAMILY and SELF.

**Table 4:** Precision, Recall, and F_1_-scores for each label on the held-out test data

Label	Precision	Recall	F_1_-score	Nr. of instances
AGE	0.797	0.505	0.618	93
AMOUNT	0.618	0.778	0.689	81
CONDITION	0.651	0.651	0.651	261
EVENT	0.511	0.630	0.564	73
FAMILY	0.706	0.859	0.775	249
INDEX	0.000	0.000	0.000	7
NEG	0.421	0.571	0.485	14
O	0.908	0.872	0.890	2066
SELF	0.929	0.730	0.818	126
TEMPORAL	0.425	0.761	0.545	67
Weighted Average (SVM)	0.835	0.821	0.824	3037
Dictionary baseline	0.770	0.607	0.647	3037
SVM (excluding “O” label)	0.678	0.712	0.684	971
Dictionary baseline (excluding “O” label)	0.543	0.720	0.581	971

We attempt to identify the mistakes of the classifier by looking at the confusion matrix in the Table 5. There is misclassification between AGE and AMOUNT, which are numbers. This happens to be the case with the categories that involve numbers such as AGE, AMOUNT, and TEMPORAL categories. The highest number of mis-classifications occur between CONDITION and EVENT labels.

**Table 5:** Confusion matrix for entity recognition experiments

AGE	AMOUNT	CONDITION	EVENT	FAMILY	INDEX	NEG	O	SELF	TEMPORAL
47	7	1	0	2	0	0	11	0	25
1	63	0	0	1	0	0	15	0	1
0	1	170	10	7	0	1	72	0	0
0	1	8	46	1	0	0	15	0	2
1	0	4	0	214	0	0	29	0	1
0	0	7	0	0	0	0	0	0	0
0	0	0	0	0	0	8	6	0	0
8	26	71	31	71	0	10	1802	7	40
0	1	0	3	7	0	0	23	92	0
2	3	0	0	0	0	0	11	0	51

During our annotation guidelines discussion, we noticed that there is no clear demarcation between CONDITION and EVENT entities. As a second experiment, we tested if the demarcation between the former categories would affect the classification of the rest of the categories by merging them under a single label. As shown in Table 6, the results do not change when we disambiguate condition and event category.

**Table 6:** Precision, Recall, and F_1_-scores for each label with CONDITION and EVENT labels merged

Label	Precision	Recall	F_1_-score	#. instances
AGE	0.810	0.505	0.623	93
AMOUNT	0.624	0.778	0.692	81
CONDITION_EVENT	0.621	0.713	0.664	334
FAMILY	0.717	0.855	0.780	249
INDEX	0	0	0	7
NEG	0.421	0.571	0.485	14
0	0.909	0.864	0.886	2066
SELF	0.929	0.722	0.813	126
TEMPORAL	0.453	0.791	0.576	67
Weighted Average (SVM)	0.837	0.823	0.826	3037
Dictionary baseline	0.771	0.613	0.650	3037
SVM (entity level)	0.685	0.734	0.698	971
Dictionary baseline (entity level)	0.543	0.720	0.581	971

### Relation extraction

In this section we report the results of our relation extraction experiments both with predicted entities and gold standard entities. The results of both the experiments are given in Tables 7 and 8. The weighted F_1_-scores for these experiments are close to the results reported in the preliminary experiments section. The use of gold standard entities improves the F_1_-scores across all the relations.

**Table 7:** Precision, Recall, and F_1_-scores for each relation with predicted entities

Relation	Precision	Recall	F_1_-score	#. instances
Holder	0.573	0.514	0.542	251
Modifier	0.558	0.36	0.438	175
No_Relation	0.766	0.859	0.81	1053
Partner	0	0	0	2
Related_to	0.748	0.608	0.671	166
Subset	0.667	0.308	0.421	13
Weighted Average	0.712	0.724	0.712	1660

**Table 8:** Precision, Recall, and F_1_-scores for each relation with gold standard entities

Relations	Precision	Recall	F_1_-score	#. instances
Holder	0.575	0.582	0.578	251
Modifier	0.67	0.417	0.514	175
No_Relation	0.79	0.856	0.821	1053
Partner	0	0	0	2
Related_to	0.772	0.693	0.73	166
Subset	0.714	0.385	0.5	13
Weighted F_1_-score	0.741	0.747	0.740	1660

The SVM classifier performs the best at ‘Related_to’ entity followed by ‘holder’ relation. The biggest improvements when using gold entity labels come with the Modifier, Related_to, and Subset class. There is an absolute improvement of 0.08 with the inclusion of gold entities. The SVM system shows a high precision with ‘Subset’ label but a low precision when using predicted entities. Both precision and recall improve when tested with gold entities.

We also report the confusion matrix for the relation labels when tested with gold entities in Table 9. Most of the mistakes occur when a relation is mis-classified as No_Relation. The partner relation is not classified correctly in both Tables 7 and 8.

**Table 9:** Confusion matrix at the relation labels classification task with gold standard labels

Holder	Modifier	No_Relation	Partner	Related_to	Subset
146	3	101	0	1	0
6	73	96	0	0	0
86	32	901	0	33	1
1	0	1	0	0	0
13	0	37	0	115	1
2	1	5	0	0	5

## Discussion

The current work is limited by the relatively modest size of the synthetic corpus, the availability of only one annotator with medical knowledge, and the use of universal dependency parsing from general Norwegian rather than clinical language. Despite these limitations, the methodology shows promise in alleviating one of the major limitations in the clinical NLP field, i.e. access to health records data.

## Conclusions

In this paper, we have described an iterative methodology for the development of annotation guidelines in concert with the production of a synthetic corpus of clinical text. A system for extraction of family history information was trained on the synthetic data and then evaluated on a small corpus of real, clinical notes, and our results indicate that the system generalizes well with only minor drops in accuracy compared to synthetic evaluation. Both the annotation guidelines and the annotated synthetic corpus have been made available, and as such constitutes the first freely available resource of Norwegian clinical text. In future work, we intend to refine the annotation guidelines regarding temporal data and important clinical entities, add further clinical annotators, and extend the validation of developed models on clinical data from other patient cohorts.

## Data Availability

The dataset supporting the conclusions of this article is available in the GitHub repository, https://github.com/ltgoslo/NorSynthClinical, DOI: 10.5281/zenodo.2667280.

## Abbreviations

CRF: Conditional Random Fields
EHR: Electronic Health Record
HCM: Hypertrophic Cardiomyopathy
IAA: Inter-Annotator Agreement
NLP: Natural Language Processing
POS: Part-of-speech
SCD: Sudden Cardiac Death
SVM: Support Vector Machine

## Footnotes

1 https://sklearn-crfsuite.readthedocs.io

2 The family terms employed here are the following lemmas: *far* ‘father’, *mor* ‘mother’, *foreldre* ‘parents’, *bror* ‘brother’, *søster* ‘sister’, *søsken* ‘siblings’, *datter* ‘daughter’, *sønn* ‘son’, *barn* ‘child’.
